# Structural and functional shifts of soil prokaryotic community due to *Eucalyptus* plantation and rotation phase

**DOI:** 10.1038/s41598-020-66004-x

**Published:** 2020-06-03

**Authors:** Douglas Alfradique Monteiro, Eduardo da Silva Fonseca, Renato de Aragão Ribeiro Rodrigues, Jacqueline Jesus Nogueira da Silva, Elderson Pereira da Silva, Fabiano de Carvalho Balieiro, Bruno José Rodrigues Alves, Caio Tavora Coelho da Costa Rachid

**Affiliations:** 1 LABEM - Laboratory of Biotechnology and Microbial Ecology, Institute of Microbiology Paulo de Góes, Department of General Microbiology, Federal University of Rio de Janeiro, Rio de Janeiro, Brazil; 2 Embrapa Soils, Rio de Janeiro, Brazil; 3 UFF – Fluminense Federal University, Rio de Janeiro, Brazil; 4 Embrapa Agrobiology, Seropédica, Brazil

**Keywords:** Soil microbiology, Element cycles

## Abstract

Agriculture, forestry and other land uses are currently the second highest source of anthropogenic greenhouse gases (GHGs) emissions. In soil, these gases derive from microbial activity, during carbon (C) and nitrogen (N) cycling. To investigate how *Eucalyptus* land use and growth period impact the microbial community, GHG fluxes and inorganic N levels, and if there is a link among these variables, we monitored three adjacent areas for 9 months: a recently planted *Eucalyptus* area, fully developed *Eucalyptus* forest (final of rotation) and native forest. We assessed the microbial community using 16S rRNA gene sequencing and qPCR of key genes involved in C and N cycles. No considerable differences in GHG flux were evident among the areas, but logging considerably increased inorganic N levels. *Eucalyptus* areas displayed richer and more diverse communities, with selection for specific groups. Land use influenced communities more extensively than the time of sampling or growth phase, although all were significant modulators. Several microbial groups and genes shifted temporally, and inorganic N levels shaped several of these changes. No correlations among microbial groups or genes and GHG were found, suggesting no link among these variables in this short-rotation *Eucalyptus* study.

## Introduction

The greenhouse effect is a natural process responsible for the maintenance of Earth’s mean temperature. Greenhouse gases (GHGs) absorb solar radiation and trap it in Earth’s atmosphere, which increases the planet’s heat budget^1^. Atmospheric increases of 40% in carbon dioxide (CO_2_), 150% in methane (CH_4_) and 20% in nitrous oxide (N_2_O) were observed from 1750 to 2011^2^. The increase in the concentrations of these gases is now causing ecological issues^3^ and extreme weather and climate events^1^.

Even though they are lower in atmospheric concentrations, CH_4_ and N_2_O have 28 and 265 times the global warming potential of CO_2_, respectively, in a 100-year period. They contribute, respectively, to approximately 17% and 6% for the positive radiative forcing of the GHG^4^. N_2_O also acts as the main ozone-depleting particle in the stratosphere^5^.

Agriculture, forestry and other land uses represented 23% of global GHG emissions from 2007 to 2016. The emissions during this period consisted of 13% CO_2_, 44% CH_4_ and 82% N_2_O^6^. Land use change currently contributes to 44% of the GHG emissions in Brazil, mainly through deforestation^7^. Brazil currently ranks sixth in global emissions^8^.

The increasing demand on raw material has driven deforestation, which has caused the decrease of 129 million hectares of the global forest area in a 25-year period, especially in South America^9^. Planted forests are an alternative for this demand, since they supply materials including wood pulp, charcoal, and lumber for industry. In Brazil, planted forests cover 7.83 million hectares. These forests are responsible for 1.3% of the country’s gross income and the planted trees are a stock of approximately 1.7 billion tons of CO_2_ equivalent. As mandated by Brazilian law, the involved companies conserve 5.6 million hectares of native land, which stocks approximately 2.5 billion tons of CO_2_ equivalent^10^.

The genus *Eucalyptus* comprises approximately 73% of the planted forests in Brazil. They are preferred because of their fast growth, high productivity and adaptation to many regions, as revealed from studies of *Eucalyptus* silviculture^11–13^. Soils from *Eucalyptus* plantations behave as atmospheric sinks for CH_4_ and as sources of CO_2_ and N_2_O^14–18^. However, as they occupy marginal soils, with low fertility (and fertilisation regime) and high acidity, the fluxes could be considered low compared with other ecosystems^19^. The short-rotation plantation has two growth phases in terms of nutrient demand and cycling: a juvenile phase up to canopy closure and another phase up to harvest^20^. How the plantation phase affects the dynamics of GHG fluxes is unclear.

Microorganisms play an important role in the emission and removal of GHGs by soils, as they cycle nitrogen (N) and carbon (C) molecules in soil environments^21^. Soil CH_4_ is produced under anaerobic conditions by methanogenic archaea and is consumed under aerobic conditions by methanotrophic bacteria^22^. Release of N_2_O from soils mainly derives from the escape of this molecule during nitrification or denitrification steps of the N cycle^23^. Understanding the correlation among specific microbial groups or the abundance of functional genes with underlying abiotic factors (*e*.*g*., temperature, humidity, nutrients, vegetation, land cover and land use)^24^ linked with GHG fluxes could lead to the development of microbial indicators that correctly assess these processes, and development of mitigation options^25^.

A previous study evaluated how *Eucalyptus* logging impacts soil microbial communities and GHG fluxes^18^. Significant changes in soil bacterial community structure and the abundance of specific genes suggested that forestry management interferes with microbial communities in the short-term. However, this study involved a single sampling, and provided no information on the impact in the longer-term. The present study was undertaken to clarify how: i. the growth period, ii. land use change and iii. seasonality impact the GHG fluxes and inorganic N levels in tropical *Eucalyptus* planted forests. Additionally, we investigate how these variables modulate microbial communities and whether there is a link between the microbial community and the GHG fluxes or the inorganic N levels.

To accomplish these goals, we selected three adjacent areas: a recently logged *Eucalyptus* forest area with 1-month seedlings (juvenile phase), a fully developed *Eucalyptus* forest with 6-year old trees near the end of the rotation cycle and a native forest area. We surveyed these areas for 9 months, sampling for GHG and inorganic N levels, and used 16 S rRNA gene sequencing and quantitative polymerase chain reaction (qPCR) of key genes involved in the CH_4_ and N cycles to examine the research questions. Our hypothesis was that key microbial groups and gene abundances would correlate with GHG fluxes in a 9-month period, and that the land use or the *Eucalyptus* trees growth phase would recruit specific microbial groups.

## Methods

### Sampling area

All samplings were performed in an area belonging to the Celulose Nippo-Brasileira (CENIBRA) company, located in Belo Oriente, Minas Gerais State, Brazil (19°18′54′S, 42°23′48′W; 300 m altitude). The company has rotated *Eucalyptus* plantations in the area since 1960, with a 7 to 9-year period of tree growth before harvesting. The planted seeds are clones of *Eucalyptus urograndis*, a hybrid from *E. grandis* and *E. urophylla*. In compliance with Brazilian law, a section of native forest is maintained inside the company’s area.

The predominant vegetation of the region is the Atlantic forest. The climate is defined as Aw (tropical with a dry winter) according to the Köppen climate classification. The annual mean temperature varies from 22 °C to 27 °C, and the annual mean precipitation varies from 701 to 1,500 mm. The landscape comprises a high slope of 26°. To avoid the effect caused by the differences in relation to the slope, we divided the area into 4 quartiles, perpendicular to the direction of the slope. Samples were taken over the entire length of the second quartile (from top to bottom).

The soil is defined as red-yellow Ferralsol (high metal oxides contents, low fertility, and a medium to a loamy texture). The physical-chemical contents of the soil were previously measured^18^ (Supplementary Table 1).

### Experimental design

To understand both the effects of *Eucalyptus* establishment and its growth phase (juvenile and adult) as compared to native forest soils, an area undergoing *Eucalyptus* rotations since 1978 was chosen for study. In 2017, trees in this area were 6-years-old, and approximately half of them were logged and seedlings were planted. The study area was divided into three treatments. The first was an area of adult (6-years-old) *Eucalyptus* (OE), representing a plantation at its last management year. This area contained 470 trees planted in rows and spaced by 3 × 2.5 m. At the end of the sampling, the trees were 7-years-old and ready to be harvested. At this stage (end of rotation), a large mass of litterfall (organic matter and nutrients) has returned to soil and is mineralized, representing the key process to providing nutrients to the stand.

The second area was young *Eucalyptus* (YE). It was a 1-month-old seedlings area, planted approximately 1 week after *Eucalyptus* logging, representing a standard *Eucalyptus* forest renewal. This area contained 680 seedlings planted in rows and spaced by 3 × 2.5 m. At the end of the sampling, the trees were 10-months-old and approximately 2.5 m height. At this stage, crop residues are the main supplier of nutrients to plants.

The third area was native forest (NF). It was an Atlantic forest remnant maintained by CENIBRA, representing a closer condition to the region’s original state.

To check for temporal shifts, four campaigns for GHG sampling, four for inorganic N content sampling and two for microbiological soil sampling (beginning and end of the period) were performed in March 2017 (summer, wet season; Time 1), June 2017 (fall, dry season; Time 2), September 2017 (winter, dry season; Time 3) and December 2017 (spring, wet season; Time 4) (Supplementary Fig. 1).

### GHG quantification

To quantify N_2_O and CH_4_ fluxes, five closed static chambers were deployed in each area. The static chamber design was previously described^26^. The chambers had a steel frame base (40 cm × 60 cm), mounted at a depth of 6 to 7 cm, 20 cm from a randomly chosen *Eucalyptus* tree, seedling or a NF tree. The base was left in the same position during the whole experiment. Polyethylene lids were attached and sealed to the base with soft rubber and covered with a foam layer and a reflective adherent mantle. The lid of the mounted chamber was approximately 13 cm above the soil surface. A three-way-tap at the lid permitted 30 mL gas samples to be withdrawn from inside the chambers with a polypropylene syringe. The syringe air was transferred to 20 mL chromatography vials that been previously depressurized close to −100 kPa. Sampling was performed 0, 20, 40 and 60 min after chamber closure, always between 8 a.m. and 10 a.m.^26^.

Gas flux quantification was carried out by gas chromatography using a GC 2014 apparatus (Shimadzu, Japan). Soil N_2_O and CH_4_ fluxes were calculated based on analytical curves of standards. The fluxes were used to transform the integrated area of each sample peak into gas concentrations. The flux (*F*) was calculated as:

$$F=({\rm{\delta }}{\rm{C}}/{\rm{\delta }}{\rm{t}})\times (V/A)\times M/Vm$$

where δC/δt is the slope of a linear function fitted to the gas concentration of samples, V is the volume (L) of the chamber, A is the area covered by the chamber in m^2^, M is the molecular weight and Vm is the molecular volume at the sampling temperature.

### Inorganic N quantification

Four soil samples were randomly collected inside the three areas. Collection was done at the beginning and at the end of the sampling campaign to quantify nitrate (NO_3_^−^) and ammonium (NH_4_^+^) in soil extracts. The mineral N content was extracted from 20 g of fresh soil with 60 mL of 2 M KCl after 1-h of rotary shaking at 220 rpm, and the supernatant was filtered^27^. The resultant solution was used to determine NO_3_^−^ by ultraviolet spectrometry and NH_4_^+^ by salicylate reaction^28^. The arithmetic mean of the four values was used for both contents.

### Soil samples for microbial analysis

Five soil samples were taken per area in each sampling time approximately 10 cm apart from the different gas flux chambers. Each point was considered as a replicate. At each sampling time a 1.5 cm diameter steel tube probe that had been previously sterilised at 180 °C for 3 h to remove contaminants, especially nucleases, was inserted approximately 7 cm into the soil. Collected soil was deposited in a sterile 50 mL propylene tube, mixed and subdivided into two subsamples. Samples were frozen in liquid nitrogen in the field and maintained until DNA or RNA extraction.

### DNA and RNA extraction

DNA was extract from approximately 500 mg of each soil sample using the Fast DNA Spin Kit for Soil (MP Biomedicals, USA). The DNA was purified using the NucleoSpin Soil Kit (Macherey-Nagel, Germany) from the sixth step of its extraction protocol, due to residual presence of humic acids after the final step of extraction.

RNA was extracted from approximately 2 g of soil using the RNA Power Soil – Total RNA Isolation Kit (Mobio, USA) according to the manufacturer’s protocol. After the RNA extraction, 7 μL were treated with RQ1 RNase-Free DNase (Promega, USA) to remove any DNA contamination.

Nucleic acid purity and concentration was assessed using a NanoDrop 1000 device (Thermo Fisher Scientific, USA) and a Qubit 3.0 fluorometer (Thermo Fisher Scientific, USA), respectively.

### 16S rRNA gene sequencing and analysis

DNA extracted from soil samples was examined using 16S rRNA gene sequencing to understand shifts in bacterial communities due to land use, *Eucalyptus* growth phase, and temporality. Soil samples from time 1 had their sequencing performed as previously described^18^. Time 4 soil samples were sequenced by the StarSeq Company (www.starseq.com, Germany) on MiSeq equipment using paired-end runs (2 × 250) according to the manufacturer’s guidelines. The primers used were 515FB (GTG YCA GCM GCC GCG GTA A)^28^ and 926 R (CCG YCA ATT YMT TTR AGT TT)^29^. These primers target the V4-V5 regions of the 16S rRNA gene. Sequencing error tax rate was assessed by the coincident use of the ZymoBIOMICS Microbial Community DNA Standard (Zymo Research, USA) with the samples. The error tax rate from this sequencing was 0.08% per base, as assessed by the positive control.

Bioinformatics analysis were done using Mothur software v. 1.41.3^30^. Forward and reverse paired sequences were grouped into contigs and their barcodes and primers were removed from sequences. Sequences containing ambiguities (N-base) or containing more than 8-mer homopolymers were removed. All sequences presenting inconsistent sizes with what was expected for the amplicon were also removed. Unique sequences were grouped through the unique.seqs command. A virtual PCR was done in the Silva database^31^ using the 515FB and 926 R primers. The sequences were then aligned with the database. Badly aligned sequences and non-informative columns were eliminated. All sequences were trimmed to fully overlap and unique sequences were again grouped. Pre-clustering of the sequences with a difference threshold of 2 bp was done. The chimeras were checked and removed using the chimera.vsearch command^32^. Virtual PCR was performed on the Ribosomal Database Project^33^ using the 515FB and 806RB (GGA CTA CNV GGG TWT CTA AT)^34^ primers for the V4 hypervariable region (in common among time 1 and 4 sequencings). The resulting reference file was used to classify our sequences using an 80% bootstrap threshold. Sequences from mitochondria, chloroplasts, Eukarya, Archaea and unknown domain were removed. Sequences from our samples that matched those at the negative control were also removed. In addition, OTU clustering was performed with a 3% similarity cutoff and singletons were removed. We normalized all samples based on the size of the smallest one (16,745 sequences) by random subsampling. Rarefaction curves, alpha diversity indexes, relative abundance of taxa, and an OTU distribution matrix were exported from the software.

To assess archaeal community structure, an exact sequence variant (ESV) clustering methodology was carried out using the Deblur algorithm^35^ in Mothur v1.41.3 during the pre-clustering step. After chimera removal, our sequences were classified according to the RDP database. Sequences from mitochondria, chloroplasts, Eukarya, Bacteria and unknown domain sequences were removed. ESVs from unique sequences were then removed. Because of the low number of sequences after the error correction steps, we had four samples removed at our subsample step (OE1.4, OE3.4, NF1.4, YE2.4; <225 sequences).

Raw sequence data were deposited in the NCBI Sequence Read Archive (SRA) and are available under Bioproject accession numbers PRJNA471919 (time 1) and PRJNA591370 (time 4).

### RT and qPCR reactions

The construction of the standard curves and the qPCR reactions of the time 1 samples were performed as previously described^18^, except for *nir*S and *pmo*A genes.

Time 4 samples were analysed by qPCR for the selected genes using the GoTaq qPCR Master Mix (Promega, USA) on extracted DNA, and were quantified with the QuantStudio 3 device (Applied Biosystems, USA) using the SybrGreen excitation setting. The analyses were performed with the QuantStudio Design & Analysis Software v1.4.3 (Applied Biosystems, USA). Each reaction was 12 µL and contained 2 µL DNA, 0.48 µL (0.4 µM) of each primer, 0.24 µL of formamide (2%; for *nir*S and *nir*K reactions only), 6 µL of GoTaq qPCR Master Mix (2×) and nuclease-free water to the final volume of 12 µL. All the reactions were performed in triplicate along with a -RT control (without reverse transcription, for RT-qPCR only), eight plasmid dilutions (ranging from 10^9^ to 10^2^ copies), and a no-template control (NTC) in a MicroAmp Optical 96-Well Reaction Plate (Thermo Fisher Scientific, USA). The following protocol (fast setting) was used: 95 °C for 20 s; 40 cycles of 95 °C for 3 s, annealing temperature (Supplementary Table 2) for 20 s and 72 °C for 45 s; 95 °C for 1 s, 60 °C for 20 s and 95 °C for 1 s (melting curve analysis). Fluorescence was read during the elongation step of each cycle.

RT-qPCR reactions were done using the GoTaq 2-Step RT-qPCR System kit (Promega, USA) with the same protocol as for qPCR of time 4 samples.

Absolute quantifications based on the standard curve created with the plasmid dilutions were performed. The quantified number of copies were normalised to a nanogram of extracted RNA and to a gram of soil for DNA. Reactions efficiencies were calculated as:

$$E=-1+{10}^{\left(-\frac{1}{slope}\right)}$$

and quantities were normalised as gene/16S ratio to minimize extraction bias.

### Statistical analyses

The gene/16S ratios, N_2_O and CH_4_ fluxes, NO_3_^−^ and NH_4_^+^ measurements, alpha diversity indexes and relative abundances of bacterial taxa were tested for differences among treatments by two-way analysis of variance (two-way ANOVA) using treatment and time of sampling as independent variables, followed by Tukey’s post hoc test. All data were checked for normality of distribution by Shapiro-Wilk’s test and homoscedasticity among treatments by the Levene test. If the data failed both assumptions, a Box-Cox transformation was executed. For GHG and inorganic N plots, the standard error of the mean (SEM) was used instead of the standard deviation (SD). Spearman’s correlations among gene/16S ratios and the 49 most abundant bacterial OTUs (>0.5% relative abundance) with gas fluxes and inorganic N contents were generated and the p-values were Bonferroni corrected.

A non-metric multidimensional scaling (nMDS) ordination was performed from the OTU distribution of treatments with the Bray-Curtis dissimilarity index and using GHG fluxes and inorganic N contents as correlating parameters. A two-way permutational multivariate analysis of variance (two-way PERMANOVA) followed by Bonferroni correction for p-values was performed, to test for the impact of time and treatments on OTU distribution. All statistical tests were done using Past3.24 software^36^.

We then performed a blocked Indicator Species Analysis^37^ based on the 49 most abundant bacterial OTUs (>0.5% relative abundance) using the following parameters: YE × OE and YE + OE × NF. Only the OTUs that were significantly impacted (p < 0.05) and had an indicator value > 60 are demonstrated. This analysis was conducted with PC-ORD 6.0 software^38^.

For all boxplots, whiskers represent the minimum and maximum values and box the interquartile range (Q1-Q3, line representing Q2, *i*.*e*., the median).

## Results

### GHG fluxes and inorganic N contents

**Figure 1:**
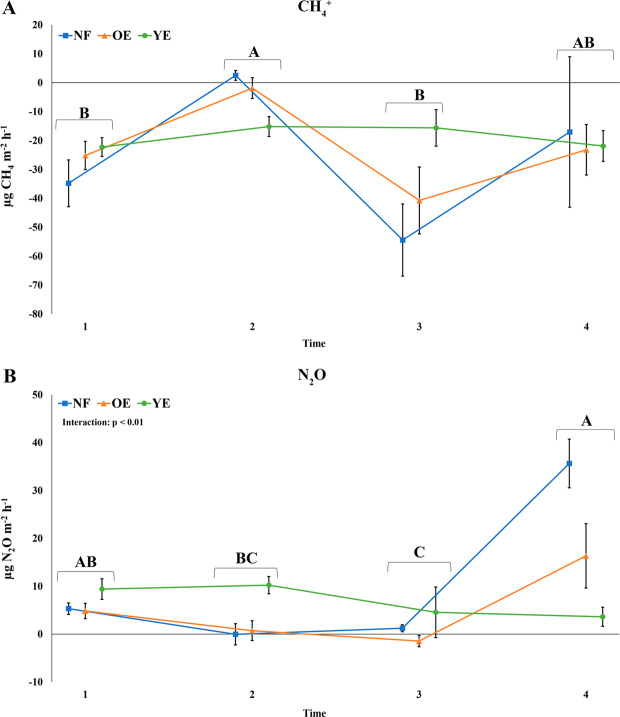
GHG fluxes (**A**: CH_4_; **B**: N_2_O) in the native forest (NF), old *Eucalyptus* (OE), and young *Eucalyptus* (YE) areas at times 1 (March), 2 (June), 3 (September), and 4 (December). Values represent means with the vertical error bars denoting SEM. Statistical differences are expressed as different upper-case letters for the time factor. No statistical differences were found for the treatment factor (two-way ANOVA followed by Tukey’s test; p < 0.05).

All areas were CH_4_ sinks and N_2_O sources during the year. A statistical difference was evident between the sampling time factor for CH_4_ and N_2_O, but not between areas (Fig. 1A,B). Time 2 (June) showed lower net negative CH_4_ fluxes from the *Eucalyptus* areas, and a slightly net positive CH_4_ flux in NF, but without statistical difference to time 4 (December). Times 1 (March) and 3 (September) showed the greatest net negative CH_4_ fluxes. For N_2_O fluxes, time 4 had the greatest means (except for YE), with no statistical difference to time 1. Times 2 and 3 had lower net N_2_O fluxes than the others (although time 2 did not differ from 1), with two events of net negative fluxes.

**Figure 2:**
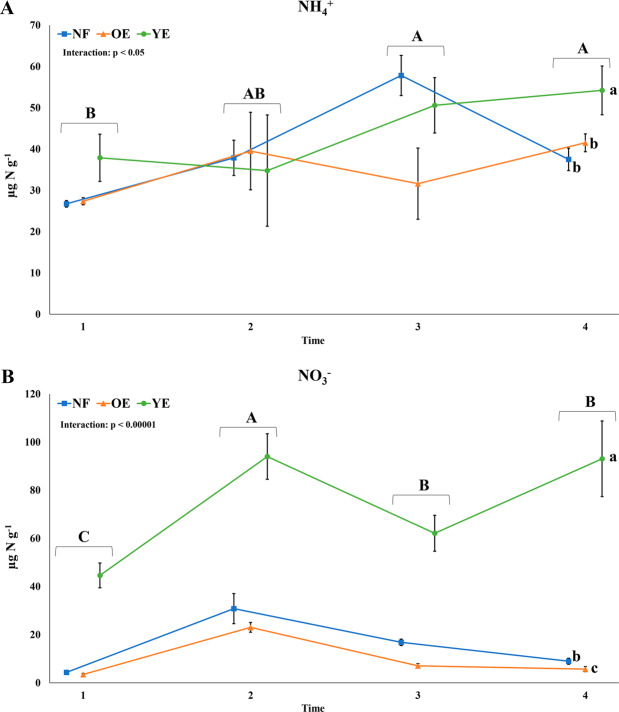
Inorganic N contents (**A**: NH_4_^+^; **B**: NO_3_^−^) found in the native forest (NF), old *Eucalyptus* (OE), and young *Eucalyptus* (YE) areas at times 1 (March), 2 (June), 3 (September), and 4 (December). Values represent means, and SEM is given as vertical error bars. Statistical differences are expressed as different upper-case letters for the time factor and as different lower-case letters for the treatment factor (two-way ANOVA followed by Tukey’s test; p < 0.05).

Regarding the inorganic N contents, NH_4_^+^ and NO_3_^−^demonstrated differences among treatments and times according to two-way ANOVA (Fig. 2A,B). Soil NH_4_^+^ concentrations seemed to increase from time 1 to 4, with times 3 and 4 differing from time 1. YE was statistically different from the other groups. For NO_3_^−^, YE had evidently higher values over the other two treatments throughout the year. NF showed higher values than OE. Time 2 showed the higher results, followed by times 3 and 4, which were not statistically different.

### Structural profile of microbial communities

A total of 502,350 sequences were obtained after the quality filtering and random subsampling, resulting in 16,745 sequences per sample. Good coverage of our samples was obtained, as evident by the rarefaction curves (Supplementary Fig. 2). We clustered the sequences into 7,754 OTUs (with a 3% dissimilarity threshold). Surprisingly, higher richness (indicated as number of OTUs) and diversity (indicated as Shannon index) values were observed inside *Eucalyptus* areas (OE and YE) than in the NF area (Table 1). Statistical testing supported the difference in means. No statistically significant difference among *Eucalyptus* treatments or between times 1 and 4 for richness and diversity were found.

**Table 1:** Bacterial alpha diversity based on the 16S rRNA gene sequencing from native forest (NF), old *Eucalyptus* (OE) and young *Eucalyptus* (YE) at time 1 (1) and time 4 (4).

Alpha diversity	NF.1	NF.4	OE.1	OE.4	YE.1	YE.4
OTU richness	1158 (143)b	1201 (139)b	1458 (131)a	1365 (129)a	1518 (225)a	1289 (66) a
Shannon index	4.93 (0.19)b	5.09 (0.14)b	5.5 (0.19)a	5.58 (0.15)a	5.61 (0.17)a	5.59 (0.09)a

Values represent the mean of five replicates, with the standard deviation shown in brackets. Statistical differences were found in the treatment factor (two-way ANOVA followed by Tukey’s test; p < 0.05) and are represented as different letters.

**Figure 3:**
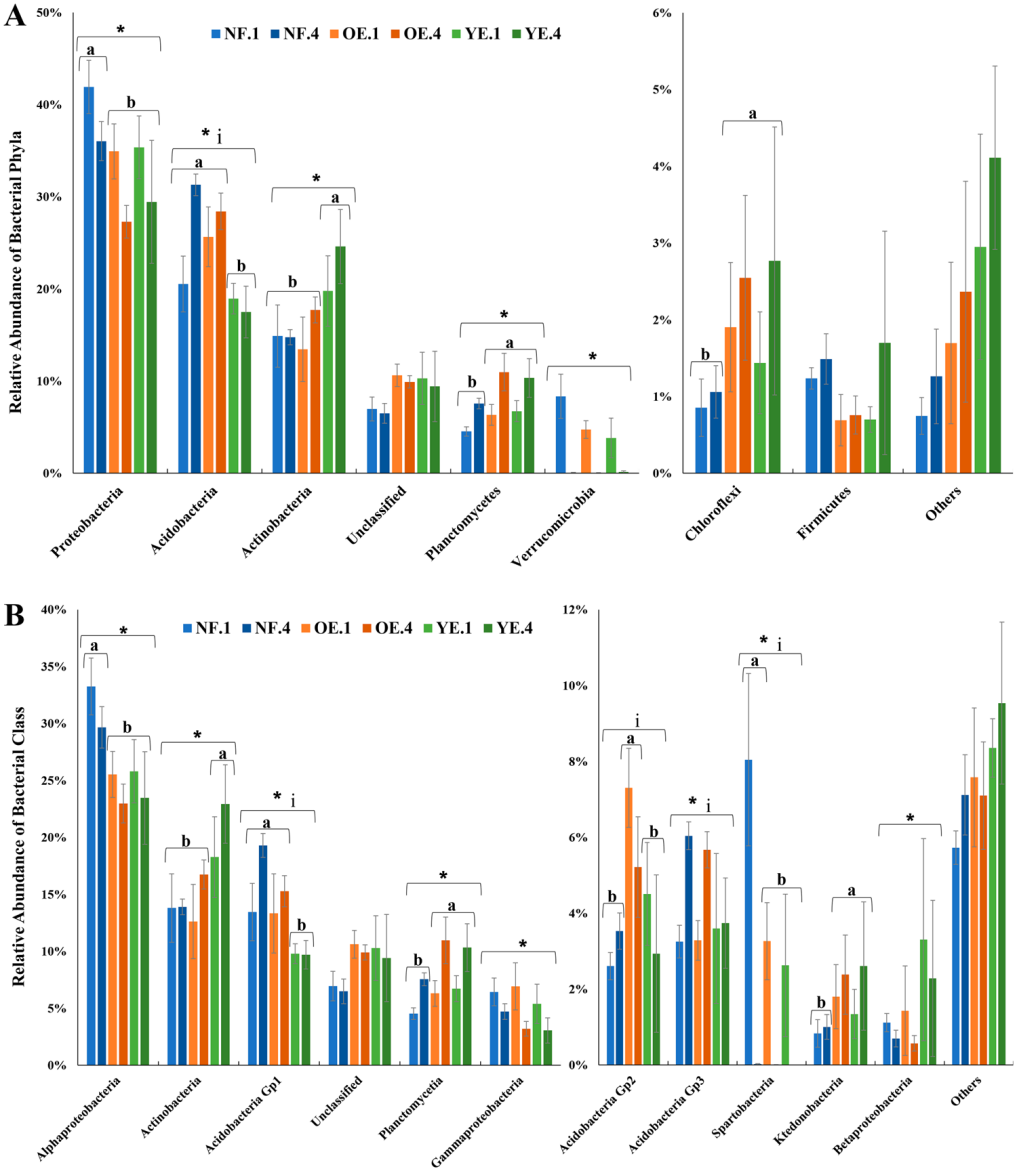
Mean relative abundance of bacterial phyla (**A**) and classes (**B**) in native forest (NF), old *Eucalyptus* (OE), and young *Eucalyptus* (YE) areas at time 1 (1) and time 4 (4). SD is denoted by the vertical error bars. Statistical differences (two-way ANOVA followed by Tukey’s test; p < 0.05) among treatments are represented as different letters, differences among times 1 and 4 by asterisks, and interactions among factors by the letter i. Taxonomies are given based on the RDP database with a bootstrap value of 80%.

Regarding the bacterial composition at the phylum level, the following taxa were shared among all treatments: Proteobacteria, Acidobacteria, Actinobacteria, Planctomycetes, Verrucomicrobia, Chloroflexi, Firmicutes, Bacteroidetes, candidate division WPS-1, candidate division WPS-2, Gemmatimonadetes, Armatimonadetes and Nitrospirae (ordered by decreasing mean relative abundance among all treatments). Unclassified sequences accounted for 6.49% to 10.62% among treatments. The seven most abundant phyla constituted at least 86.47% of all sequences inside each treatment, and were chosen for the graphical plot (Fig. 3A). Time and land use (NF × OE + YE) in combination influenced the relative abundance of some phyla, including Proteobacteria, Planctomycetes, and Chloroflexi. Verrucomicrobia only differed temporally, and Acidobacteria and Actinobacteria were different among time and seemed to have been impacted by *Eucalyptus* growth (NF and OE × YE). Firmicutes displayed no statistical difference among treatments. An interaction among factors was found for Acidobacteria.

Twenty-six classes were shared among all treatments. The ten most abundant classes constituted at least 81.05% of the sequences of each treatment (Fig. 3B). Gammaproteobacteria, Gp3, and Betaproteobacteria were influenced only by time. Alphaproteobacteria, Planctomycetia, and Spartobacteria were affected by time and land use (NF × OE + YE). Time and *Eucalyptus* growth period (NF + OE × YE) differentiated the relative abundance of Actinobacteria and Gp1. Land use alone impacted Ktedonobacteria. Gp2 displayed a difference in OE compared to the other areas. An interaction among factors was found for Gp1, Gp2, Gp3, and Spartobacteria.

**Figure 4:**
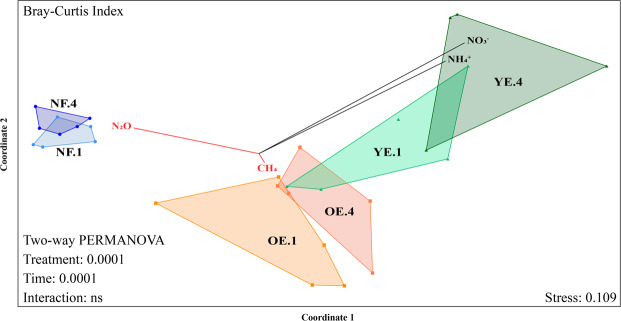
Non-metric multidimensional scaling (nMDS) ordination with Bray-Curtis dissimilarity index of OTU distribution in native forest (NF), old *Eucalyptus* (OE), and young *Eucalyptus* (YE) areas at time 1 (1) and time 4 (4). Red lines represent gas fluxes plotted as vectors. Black lines represent inorganic N. A two-way PERMANOVA test was performed using time and treatment as factors.

According to the nMDS, *Eucalyptus* areas shared a more similar bacterial community distribution than with NF, despite their high variability (Fig. 4). NF areas showed a lower dispersion among samples, which indicated lower beta-diversity and higher stability compared to *Eucalyptus* areas. Whereas the NF community structure remained very similar from time 1 to 4, YE samples showed high variability and, at time 4, the difference in structure was more pronounced to OE than it was just after cutting. Two-way PERMANOVA test revealed that both treatment and time factors induced statistical differences among communities, without interaction among the factors. Soil N_2_O fluxes vector correlated with NF samples, while inorganic N contents correlated with YE.4 area.

**Figure 5:**
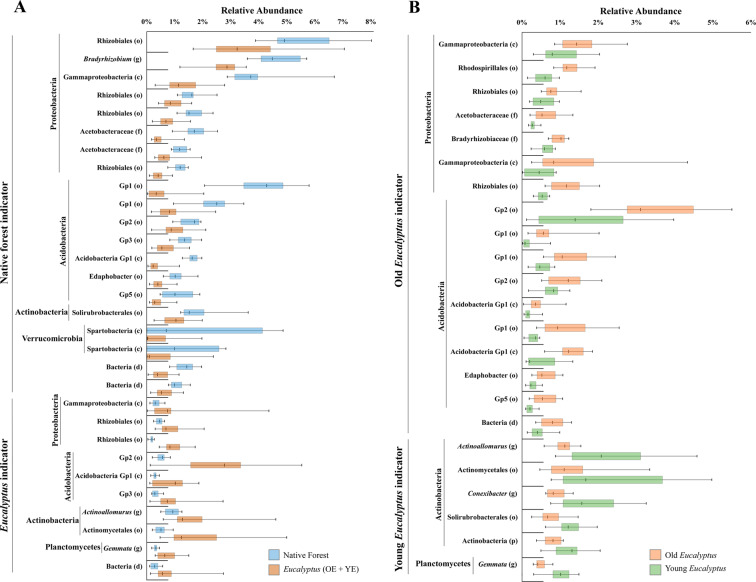
Disparity of relative abundance from the most abundant OTUs among *Eucalyptus* areas versus native (**A**), and old *Eucalyptus* versus young *Eucalyptus* (**B**). OTUs were submitted to a blocked Indicator Species Analysis (ISA). Only those that were significantly different are represented. The finest taxonomy is given (d – domain, p – phylum, c – class, o – order, f – family, g – genus).

A further analysis of the 49 most abundant OTUs (those with a relative abundance of at least 0.5%) using a blocked Indicator Species Analysis (ISA) was performed. These 49 OTUs represented 52% of the global relative abundance. The analysis revealed that 30 OTUs were impacted by land use (NF × OE + YE), with 20 significantly more abundant in NF areas and 10 in *Eucalyptus* areas. Regarding *Eucalyptus* growth phase effect (OE × YE), 23 OTUs were impacted, with 17 associated with the OE area and six with the YE area. The affected OTUs and their taxonomical affiliations are represented in Fig. 5A,B. The time effect (growth period) showed a clear pattern at the phylum level, with OE indicators belonging to Proteobacteria and Acidobacteria, while YE indicators belonged to Actinobacteria phylum and to Planctomycetes.

Regarding the Archaeal community analysis, the *Nitrososphaera* genus represented 96.7% up to 100% of the sequences in all treatments. Archaeal community showed a pattern like the bacterial community, with *Eucalyptus* areas having a more diverse and rich community than NF area (Supplementary Fig. 3).

To better understand the relationship of prokaryotic community members and our gas and soil monitored variables, Spearman correlations (n = 30) was performed among the 49 more abundant bacterial OTUs and GHG fluxes or inorganic N contents (Table 2). No correlations among the OTUs and GHG were found. Multiple correlations between bacterial OTUs and soil NH_4_^+^ and NO_3_^−^ contents were obtained. Among the positive correlations with both contents, one OTU was assigned as *Actinoallomurus* genus and one as Actinomycetales order. Three Spartobacteria OTUs negatively correlated with both mineral N forms. Members of the Proteobacteria phylum were all negatively correlated with NH_4_^+^, with one Gammaproteobacteria, four Rhizobiales, and one *Bradyrhizobium* OTU. A Bradyrhizobiaceae OTU was negatively correlated with NO_3_^−^ content. Two negative and one positive correlations were observed between representatives of the Acidobacteria phylum for NH_4_^+^ content. A positive correlation among soil NH_4_^+^ and bacterial diversity was also found (Spearman correlation: NH_4_^+^ and Shannon index, p < 0.001, r = 0.6).

**Table 2:** Spearman correlations (n = 30) among bacterial OTUs and inorganic N contents.

Parameter	Phylum	Finest taxonomy	p	R
NH_4_^+^	Actinobacteria	Actinomycetales (o)	<0.0001	0.93
		*Actinoallomurus* (g)	<0.001	0.71
	Proteobacteria	Gammaproteobacteria (c)	<0.0001	−0.88
		Rhizobiales (o)	<0.0001	−0.75
			<0.001	−0.74
			<0.001	−0.70
			<0.01	−0.67
		*Bradyrhizobium* (g)	<0.001	−0.74
	Verrucomicrobia	Spartobacteria (c)	<0.0001	−0.87
			<0.0001	−0.81
	Acidobacteria	Gp1 (o)	<0.01	−0.69
		Acidobacteria Gp1 (c)	<0.05	−0.60
		Gp3 (o)	<0.01	0.63
NO_3_^−^	Proteobacteria	Bradyrhizobiaceae (f)	<0.01	−0.68
	Actinobacteria	*Actinoallomurus* (g)	<0.01	0.67
		Actinomycetales (o)	<0.01	0.64
	Verrucomicrobia	Spartobacteria (c)	<0.05	−0.61

The table contains only the correlations with p < 0.05 (Bonferroni corrected) and r > |0.6 | (correlation coefficient). No correlations among GHG fluxes and the bacterial OTUs were found (n = 30). The finest taxonomy is given (d – domain, p – phylum, c – class, o – order, f – family, g – genus).

### Functional profiles of microbial communities

**Figure 6:**
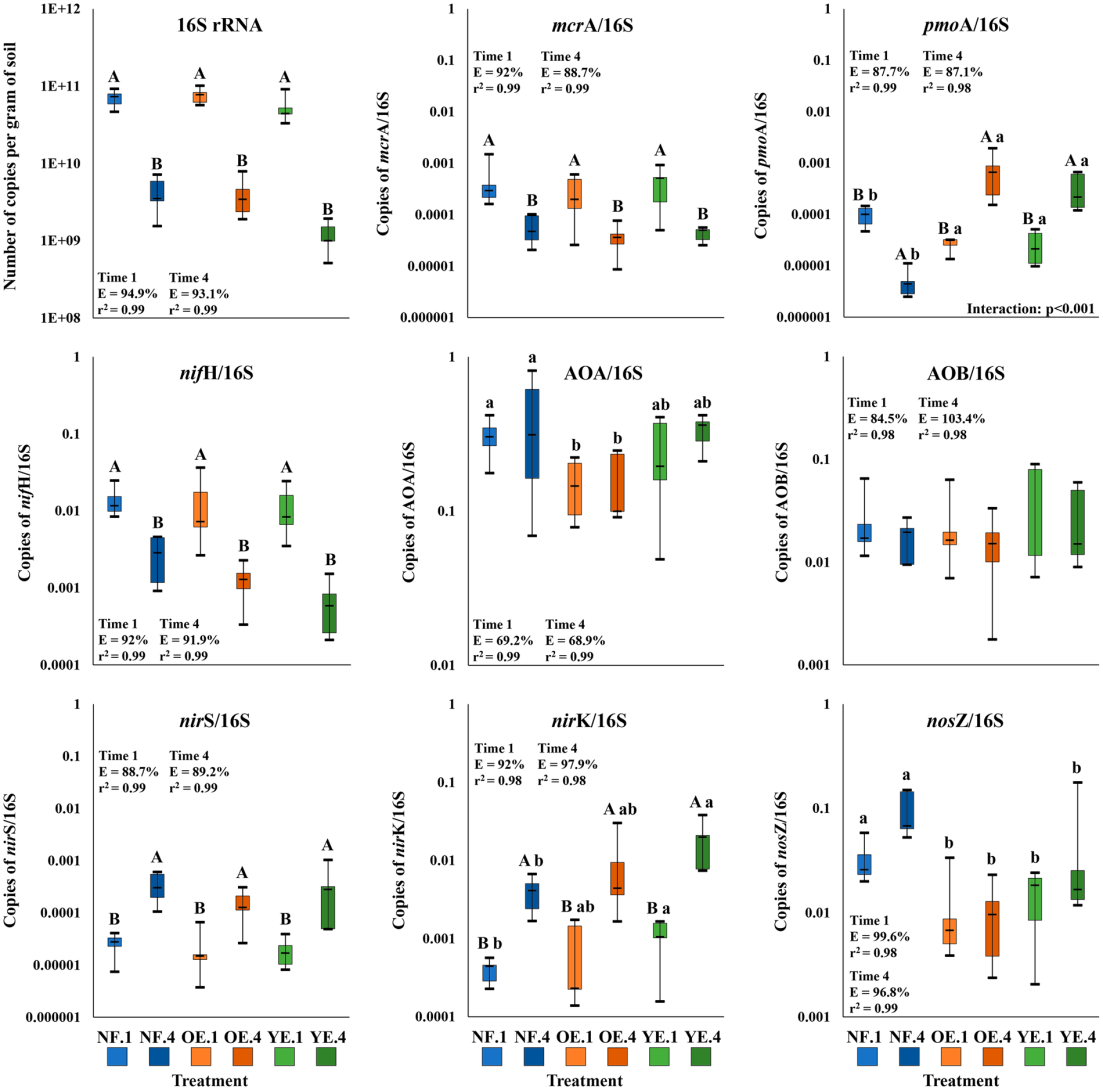
Boxplot graphs of 16S rRNA gene and gene/16S ratios quantified by qPCR in native forest (NF; blue), old *Eucalyptus* (OE; orange) and young *Eucalyptus* (YE; green) areas at time 1 (1; lighter shades) and time 4 (4; darker shades). Statistical differences are expressed as different upper-case letters for the time factor and as different lower-case letters for the treatment factor (two-way ANOVA followed by Tukey’s test; p < 0.05). Abbreviations are: *mcr*A – methyl coenzyme M reductase subunit alpha, *pmo*A – particulate methane monooxygenase subunit alpha, *nif*H – nitrogenase, AOA – ammonia-oxidising archaea, AOB – ammonia-oxidizing bacteria, *nir*S – cytochrome *cd*_1_-containing nitrite reductase, *nir*K – copper-containing nitrite reductase and *nos*Z – nitrous oxide reductase.

RT-qPCR (RNA-based) and qPCR (DNA-based) approaches were used to evaluate the microbial community metabolic activity and potential. We were unable to quantify transcripts for the genes involved in CH_4_ and N cycles, independent of the treatment or time of sample (detection limit was 10^2^, data not shown). Replicates showed inconsistency in quantification and several non-specific reactions, demonstrated by dissociation curves and gel electrophoresis, despite the good quality of RNA extracts. As an alternative, traditional qPCR was used, which enabled the assessment of the metabolic potential of the samples (Fig. 6).

All qPCRs were specific, as determined by melting curve analysis and gel electrophoresis from the products. Run data are presented in Supplementary Table 3.

Gene quantification showed that the number of copies of 16 S rRNA gene were lower at time 4 than at time 1 (from approximately 10^10^ to 10^9^ copies; Fig. 6). Ratios of *mcr*A/16S (methanogenesis), *pmo*A/16S (methanotrophy), *nif*H/16S (nitrogen fixation), and *nir*S/16S and *nir*K/16S (denitrification) were also impacted by time. The ratios of *mcr*A/16S and *nif*H/16S decreased from time 1 to 4, while the ratios of *nir*S/16S and *nir*K/16S increased. *pmo*A/16S increased from time 1 to 4 for the *Eucalyptus* treatments, but showed a decrease for NF. The treatment factor affected *pmo*A/16S, AOA/16S (nitrification), *nir*K/16S, and *nos*Z/16S (denitrification). *pmoA/16S* and *nos*Z/16S ratios were different between *Eucalyptus* (YE + OE) and NF; AOA/16S had a difference between NF and OE and *nir*K/16S among NF and YE. No statistical differences among treatments or times for the AOB/16S ratio were detected.

To evaluate if there was a link between gene copy number and the other sampled variables, we tested the number of copies of 16S rRNA gene and gene/16S ratios for correlations with GHG and N contents. Only correlations with N contents were found (Table 3). 16S rRNA gene and *nif*H/16S ratio negatively correlated with N levels in soil, while *nir*K/16S positively correlated with N content levels and *mcr*A/16S ratio negatively correlated with the quantity of NH_4_^+^ in soil.

**Table 3:** Spearman correlations (n = 30) among N contents and copies of 16S rRNA or gene/16S ratios.

N content	16 s rRNA / gene/16S ratios	p	r
NH_4_^+^	16S rRNA	<0.0001	−0.78
	*mcr*A/16S	<0.05	−0.52
	*nif*H/16S	<0.0001	−0.74
	*nir*K/16S	<0.0001	0.75
NO_3_^−^	16S rRNA	<0.001	−0.68
	*nif*H/16S	<0.05	−0.51
	*nir*K/16S	<0.01	0.58

The table contains only the correlations with p < 0.05 (Bonferroni corrected) and r > |0.5 | (correlation coefficient). No correlations among GHG fluxes and copies of 16S rRNA or gene/16S ratios were found (n = 30). Abbreviations are *mcr*A – methyl coenzyme M reductase subunit alpha, *nif*H – nitrogenase, and *nir*K – copper-containing nitrite reductase.

## Discussion

Considering all sampling times, the studied soil behaved as a CH_4_ sink and N_2_O source, as previously described for *Eucalyptus* plantations areas^14–17^ and tropical forest soils^19^. N_2_O fluxes were considerable higher at time 4 (spring; December) in the OE and NF areas. This time point was collected after an extended period of precipitation (Supplementary Fig. 1), which seemed to explain the higher emissions. Time 1 (summer; March), which did not differ statistically from time 4, was during a period of abundant precipitation but not close to a precipitation episode, whereas times 2 and 3 (fall and winter; June and September) were collected during dry periods. CH_4_ fluxes did not seem to be explained by collected environmental variables. Similar flux patterns for both gases^17^ and for N_2_O^14,39^ have been observed in planted forests. Although not statistically different, the YE area presented a smaller variation in flux in the different sampling times than the other two well-established tree areas. In other studies involving tropical rain forests, logging differentiated the GHG fluxes among sites^15,40^, probably due to an increase in soil bulk density and a decrease in air-filled spaces (YE displayed higher humidity than other areas in our study). This poorly aerated condition may favour heterotrophic and facultative anaerobic bacteria to produce different reduced derivatives of NO_3_, as NO_2_, NO and N_2_O, not only N_2_O^41^.

The lack of statistical difference in terms of GHG dynamics among the studied areas could also be explained by the high variability within treatment. The different land use did not alter most of the soil physical-chemical characteristics (Supplementary Table 1), which can correlate with GHG flux^42^. The temporal differences of GHG flux are probably the effect of pluviometry, as these soils are poor in nutrients, experience frequent water deficit, are acidic and are high in Al^+3^, which restricts microbial activity^43,44^. Increased humidity will increase microbial activity and shift the GHG dynamics.

We observed a near-zero net N_2_O flux in the NF and OE area at times 2 and 3. Some chambers showed negative fluxes, which have often been reported in the literature, and which is often linked to low NO_3_^−^ content and low O_2_ concentrations. As the contribution to N_2_O uptake, by reduction to N_2_ through the N_2_O reductase pathway is short under low supply of NO_3_, we speculate that under YE, where the nitrate concentration in soil is high and the emissions of N_2_O is also low, others factors are inhibiting the denitrification process or stimulating the N_2_O uptake, such as the change ratio of NO_3_ and N_2_O in soil profile and changes in anoxic microsite (soil structure) or even abiotic reactions of N_2_O^45–47^ (Flechard *et al*., 2005; Chapuis-Lardy *et al*., 2007; Chalk and Smith, 2020). The nitrous oxide reductase gene quantified by qPCR (Fig. 6) is clear higher in YE than OE (but not different), inside of each season, what in part could explain lower emissions of N_2_O, as cited above. Even without statistic difference, the *nirK* copies involved in nitrite reduction to NO should justify partially the low emissions of N_2_O, and probably higher of NO. The regulation mechanisms of this process are still unclear, despite of some advances^46^.

Higher inorganic N levels are expected in recently logged sites due to the decomposition of the organic matter of roots and tree residues. The NO_3_^−^ levels in soil are especially enriched in logged soils^40,48^. The lack of appreciable root depth that enables contact with and consumption of NO_3_^−^ might have contributed to the high levels throughout the sampling period. We found no correlations among GHG and inorganic N content in soils, despite the description of the correlation in other studies^16,17,49,50^.

Higher bacterial richness and diversity were observed in *Eucalyptus* areas, indicating that land use change increased these indexes. Surprisingly, this trend was observed before deforestation events^51–53^, suggesting that the alpha diversity of microbial communities increases as an adaptive response to soil disruption. We did not observe changes in alpha diversity after a 9-month period, which agrees with the theory that soil disturbance effects can persist for a long period^52,54^. We also suggest another theory, in which, paradoxically, areas of *Eucalyptus* monoculture areas harbour a more diverse microbiome when compared to the nearby Atlantic forest, probably due to plant selection or higher primary productivity^55–57^, since these areas have undergone *Eucalyptus* rotations since 1978.

Alpha diversity was previously described to be a negative indicator of land use effect, due to its high temporal variability^58^. However, presently the alpha diversity values were consistent, implicating alpha diversity as a good indicator to differentiate the *Eucalyptus* areas from NF. The land use effect over alpha diversity was also supported in another study^53^. It is important to highlight that higher alpha diversity does not necessarily imply more functional diversity in the ecosystem. In a recent study, although land use change seemed to increase 16S rRNA gene diversity, functional gene diversity was decreased in pastures compared to primary and secondary forests^59^.

Phyla composition from all treatments resembled those found in a variety of soils, including Cerrado soils^60^, *Eucalyptus* monoculture and in mixed plantations with *Acacia mangium*^56^, grasslands^61^, forests^51,53,62^, agricultural soils^53,58^, and even samples from Central Park in New York City^63^. Most phyla seem to temporally vary in abundance^53,58,64^, as soil is a complex environment that seasonally shifts in many attributes^65,66^. However, the region of study did not vary greatly in terms of temperature during the year, and displayed very distinct patterns in terms of pluviometry, which may explain the slight variances in relative abundances and community structure over time.

A recent meta-analysis included 17 studies that addressed the conversion from forest to agriculture. The findings indicated that the abundances of Proteobacteria and Acidobacteria relative are higher in natural forest soils, while Actinobacteria, Chloroflexi, and Firmicutes showed higher abundance in agricultural soils^67^. Alpha diversity showed an average increase ratio of 1.17 ± 1 fold due to land use change. We observed that Proteobacteria and Chloroflexi followed this trend. However, for Acidobacteria and Actinobacteria, OE behaved just as NF, and no differences were observed for Firmicutes. The considerable differences observed with Verrucomicrobia at times 1 and 4 were due to the near-absence of Spartobacteria class sequences at time 4.

We detected differences in beta-diversity among treatments and between times 1 and 4. Yet, land use (*Eucalyptus* plantation) seemed to impact beta-diversity more than did time and planting renewal. Both land use and management have been associated with differences in beta-diversity^68,69^, while land use alone affected beta-diversity in other studies, despite the time of sampling or land management^53,54,58^. Plant selection of the microbial community, fertilization history of *Eucalyptus* areas, soil disruption by harvesting, and differences in soil attributes could be linked to the variation in beta-diversity. It is also interesting that, after a 9-month period, YE samples were further to OE in the ordination, suggesting that it takes an even longer time for the YE microbial community to adapt to the OE structure.

Nitrogen content is correlated with many OTUs. Bradyrhizobiaceae is a family in the Rhizobiales order, which is recognized for its genera of nitrogen fixing bacteria (NFB)^70^, including *Bradyrhizobium*. Rhizobiales members were negatively correlated with N content and we found that Rhizobiales representatives were enriched in NF and OE in comparison to YE (as also seen in ISA). This is probably due to lower level of mineral N in these areas, increasing the need for higher abundance of NFB species. *Actinoallomurus* was another bacterial genus that displayed correlation with inorganic N contents. However, all known representatives of *Actinoallomurus* lack mechanisms to use inorganic N^71^. Thus, its enrichment is more likely due an indirect factor, such as higher affinity with plants present in YE areas.

It is important to highlight that all correlations must be interpreted cautiously, as they are based on multiple comparisons with data from field experiments, where many conditions cannot be controlled, increasing the chance of spurious correlations.

Presently, RNA-based qPCR was unsuccessful, However, DNA based qPCR was successfully applied. Soil is frequently an oligotrophic environment, leading to a low level of metabolism of the microbial community. This leads to a higher abundance of DNA gene copies over RNA^72–74^. The soil we studied is acidic with high Al^3+^ levels, is nutrient-poor, and has a water deficit. All these factors inhibit microbial activity. Together, these factors can explain why specific microbial populations could be detected by qPCR but not by RT-qPCR.

We found no correlations among GHG fluxes and gene abundances, even though these correlations have been described^16,75^. Temporal differences in 16S rRNA gene abundances could be explained by N enrichment (as seen by the negative correlation among 16S rRNA gene and the inorganic N content). Decreases of microbial biomass due to N fertilization have been reported^76,77^.

Methanotrophic metabolic potential differed by land use. Deforestation in Amazonian soils has been linked to decreases in methanotrophs^78^ and methane mono-oxygenase genes in these soils^59,62,78^. Although other studies reported differences in the quantity of the *mcr*A gene following deforestation^62,78^, we did not detect alterations in this gene caused by land use change.

We observed average AOA/AOB ratios from 8.3 to 19.9 in treatments. The findings support the description that archaea are the predominant ammonia-oxidizers in acidic soils^79^. It is interesting to note that our archaeal community was dominated by a single genus, *Nitrososphaera*, an AOA found abundantly in soils and some freshwater habitats^80,81^.

We detected an increase in *nir*S and *nir*K/16S ratios and a decrease in *nif*H/16S ratio from time 1 to 4, which could indicate that the community is being restructured in response to higher levels of N in these soils. The *nir*K/16S ratio positively correlated with higher levels of both NH_4_+ and NO_3_^−^, while *nif*H correlated negatively, consistent with this theory. Impacts on *nos*Z abundance by land use change were detected presently and previous studies^82–84^.

In conclusion, although no considerable differences were found among treatments, the growth phase of the young trees changed the GHG dynamics of the *Eucalyptus* area. Yet, despite *Eucalyptus* plantations are anthropically established, they showed no difference from the nearby native forest in terms of GHG fluxes in our study. Secondly, *Eucalyptus* logging substantially increased the inorganic N content of soil, which was constant over the period of our study, but this phenomenon does not drive the N_2_O emissions, probably by the harsh soil chemical conditions. On other hand, *Eucalyptus* areas displayed a richer and more diverse microbial community than the nearby Atlantic forest, which was a consistent indicator of this difference through the 9-month period studied. Land use was the main differentiating factor of the microbial community. Most taxa showed a temporal fluctuation in relative abundances, which could be shaped by the inorganic N content in the soils. Time also influenced the abundance of several genes in soils that were examined, some correlated with inorganic N contents,but it was not found correlation among assayed genes and GHG fluxes.

Planted forests in studied region have GHG emissions inhibited by the high acidity and high aluminum saturation in the soil. The decomposition of crop residues, stimulates nitrification in young eucalyptus plantations, but N_2_O emissions remained low. Changes in the structures of the communities indicated by the quantification of the number of copies of the *nir*K and *nos*Z genes, seem to be related to the low N_2_O emissions. Metanotrophy prevails over methanogenesis in both plantations and natural forests. More productive sites should be studied so that these findings can be generalized.

## Supplementary information

Supplementary information.
