# Supplementary material for: Structural and functional shifts of soil prokaryotic community due to Eucalyptus plantation and rotation phase
Source: Sci Rep. 2020 Jun 3;10:9075. doi: 10.1038/s41598-020-66004-x (PMC7270125; doi:10.1038/s41598-020-66004-x)
Supplement: Supplementary file 1 — Supplementary information. [file 41598_2020_66004_MOESM1_ESM.docx]

Structural and functional shifts of soil prokaryotic community due to *Eucalyptus* plantation and rotation phase

Douglas Alfradique Monteiro^1^, Eduardo da Silva Fonseca^1^, Renato de Aragão Ribeiro Rodrigues^2^, Jacqueline Jesus Nogueira da Silva^3^, Elderson Pereira da Silva^4^, Fabiano de Carvalho Balieiro^2^, Bruno Jose Rodrigues Alves^4^, Caio Tavora Rachid Coelho da Costa^1*^

^1^ Universidade Federal do Rio de Janeiro - LABEM - Laboratory of Biotechnology and Microbial Ecology, Institute of Microbiology Paulo de Góes, Brazil

^2^ Embrapa Solos, Rio de Janeiro, Brazil

^3^ Universidade Federal Fluminense, - UFF - Rio de Janeiro, Brazil

^4^ Embrapa Agrobiologia, Seropédica, Brazil

*** Correspondence:**caiorachid@micro.ufrj.br

**SUPPLEMENTARY MATERIAL**

Supplementary Table 1 – Soil physical-chemical characteristics of the three areas

| Soil characteristics | NF | OE | YE |
| --- | --- | --- | --- |
| Clay content (g.kg^−1^) | 600 (20) | 592 (23) | 600 (14) |
| Humidity factor (%) | 24.5 (2.86) b | 24.6 (1.49) b | 32.8 (3.78) a |
| pH (water) | 4.3 (0.15) b | 4.26 (0.05) ab | 4.00 (0.23) a |
| Total carbon (%) | 3.02 (0.90) | 3.81 (0.67) | 3.77 (1.03) |
| Total nitrogen (%) | 0.22 (0.04) | 0.21 (0.04) | 0.22 (0.04) |
| C:N ratio | 13.3 (1.62) b | 17.5 (0.76) a | 16.9 (1.65) a |
| Available P (mg.kg^−1^) | 3 (1) | 7 (1) | 19 (23) |
| N:P ratio | 663 (98.7) b | 312 (86.7) a | 221 (109) a |
| H^+^ + Al^3+^ (cmolc.dm^−3^) | 14.5 (1.8) | 16 (1.1) | 16.3 (2.2) |
| Ca^2+^ (cmolc.dm^−3^) | 0.31 (0.12) b | 0.38 (0.07) b | 0.95 (0.47) a |
| Mg^2+^ (cmolc.dm^−3^) | 0.28 (0.04) a | 0.14 (0.01) b | 0.29 (0.1) a |
| Al^3+^ (cmolc.dm^−3^) | 1.57 (0.26) | 2.06 (0.22) | 1.86 (0.41) |

Values represent the mean of five replicates (except for humidity, where n = 4). The standard deviation is in the brackets. Statistical differences (ANOVA followed by Tukey’s test; p < 0.05) are represented as different letters. Total C and N are expressed as percent weight.

Supplementary Table 2 – Genes, primers, protocols, and references used for qPCR and PCR

| Genes | **Primers names** | **Primer sequence forward / reverse (5´-3´)** | **Fragment size (bp)** | | **Annealing temperature** | **References** |
| --- | --- | --- | --- | --- | --- | --- |
| 16S rRNA | 341f/534r | CCTACGGGAGGCAGCAG / ATTACCGCGGCTGCTGG | 193 | 53°C | | [83,84] |
| *mcr*A | qmcrAf/qmcrAr | TTCGGTGGATCDCARAGRGC / GBARGTCGWAWCCGTAGAATCC | 140 | 58°C | | [85,86] |
| *pmo*A | f326/r643 | TGGGGYTGGACCTAYTTCC / CCGGCRCRACGTCCTTACC | 358 | 53.5°C | | [87] |
| *nif*H | PolF/PolR | TGCGAYCCSAARGCBGACTC / ATSGCCATCATYTCRCCGGA | 360 | 55°C | | [88] |
| Archaeal *amo*A | 19F/CrenamoA616r48x | ATGGTCTGGCTWAGACG / GCCATCCABCKRTANGTCCA | 624 | 55°C | | [89,90] |
| Bacterial *amo*A | amoA1F/amoA2R | GGGGTTTCTACTGGTGGT / CCCCTCKGSAAAGCCTTCTTC | 491 | 55°C | | [91] |
| *nir*S | cd3aF/R3cd | GTSAACGTSAAGGARACSGG / GASTTCGGRTGSGTCTTGA | 425 | 59°C | | [92] |
| *nir*K | F1aCu/R3Cu | ATCATGGTSCTGCCGCG / GCCTCGATCAGRTTGTGGTT | 473 | 62°C | | [92,93] |
| *nos*Z | nosZ1F/nosZ1R | WCSYTGTTCMTCGACAGCCAG / ATGTCGATCARCTGVKCRTTYTC | 259 | 62°C | | [94] |

Supplementary Table 3 – Description of qPCR standard curves and no-template controls

| Gene | Efficiency | r^2^ | Y-Intercept | Slope | No-template controls |
| --- | --- | --- | --- | --- | --- |
| 16S.1 | 94.9% | 0.99 | 43.177 | -3.45 | Not detected |
| 16S.4 | 93.1% | 0.99 | 39.135 | -3.5 | Not detected |
| *mcr*A.1 | 92% | 0.99 | 46.54 | -3.537 | Not detected |
| *mcr*A.4 | 88.7% | 0.99 | 43.318 | -3.625 | Not detected |
| *pmo*A.1 | 87.7% | 0.99 | 44.096 | -3.656 | Not detected |
| *pmo*A.4 | 87.1% | 0.98 | 46.992 | -3.676 | Not detected |
| *nif*H.1 | 92% | 0.99 | 43.34 | -3.531 | Not detected |
| *nif*H.4 | 91.9% | 0.99 | 44.044 | -3.532 | Not detected |
| AOA.1 | 69.2% | 0.99 | 54.61 | -4.374 | Not detected |
| AOA.4 | 68.9% | 0.99 | 46.603 | -4.394 | Not detected |
| AOB.1 | 84.5% | 0.98 | 53.448 | -3.757 | Not detected |
| AOB.4 | 103.4% | 0.98 | 50.099 | -3.243 | Not detected |
| *nir*S.1 | 88.7% | 0.99 | 40.721 | -3.627 | Not detected |
| *nir*S.4 | 89.2% | 0.99 | 42.176 | -3.610 | Not detected |
| *nir*K.1 | 92% | 0.98 | 36.82 | -3.536 | Not detected |
| *nir*K.4 | 97.9% | 0.98 | 38.642 | -3.374 | Not detected |
| *nos*Z.1 | 99.6% | 0.98 | 44.69 | -3.332 | Not detected |
| *nos*Z.4 | 96.8% | 0.99 | 44.156 | -3.4 | Not detected |

*mcr*A – methyl coenzyme M reductase subunit alpha. *pmo*A – particulate methane monooxygenase subunit alpha. *nif*H – nitrogenase Fe protein.AOA – ammonia-oxidizing archaea. AOB – ammonia-oxidizing bacteria. *nir*S – cytochrome *cd*_1_-containing nitrite reductase. *nir*K – copper-containing nitrite reductase. *nos*Z – nitrous oxide reductase


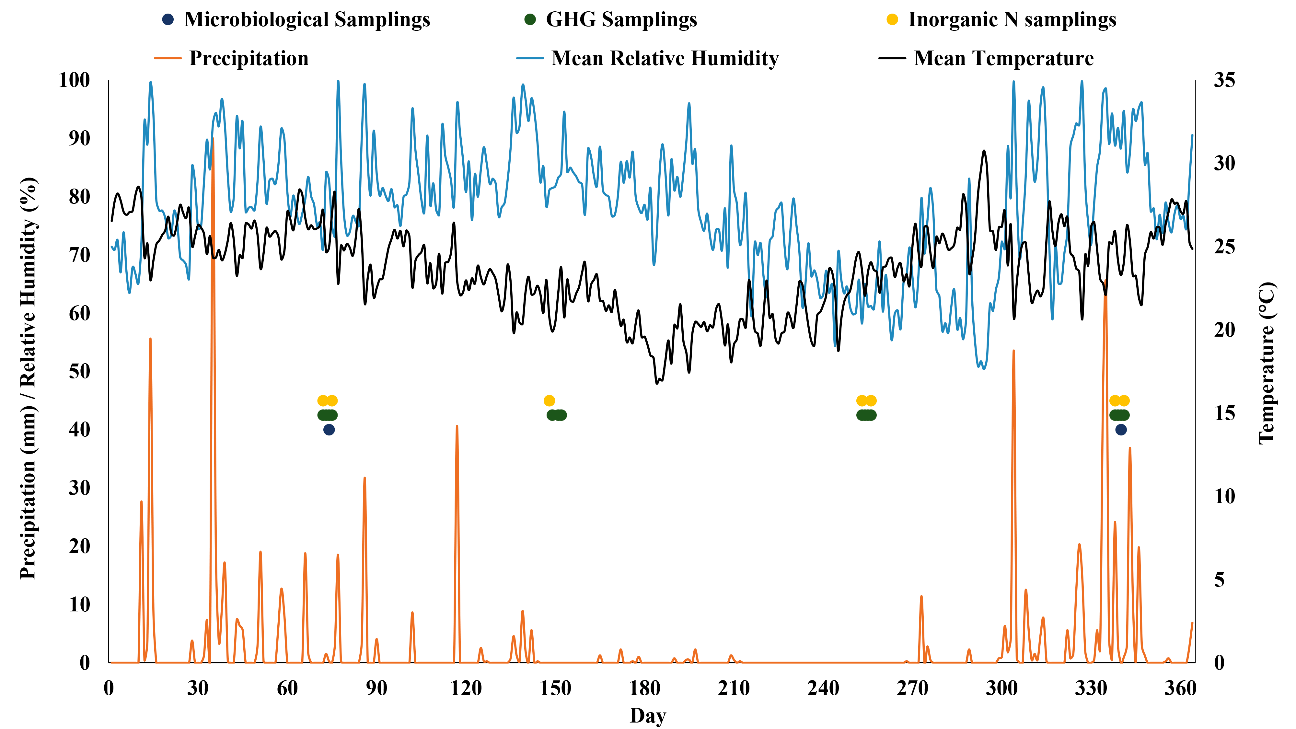


Supplementary Figure 1 – Graphical demonstration of environmental variables and sampling dates. The environmental variables values during 2017 were obtained by the weather station located inside the CENIBRA factory. Precipitation (mm; orange line), mean relative humidity (%; light blue line), mean air temperature (ºC; black line), microbiological samplings (dark blue dot), GHG samplings (dark green dots) and inorganic N samplings (yellow dots) are demonstrated.


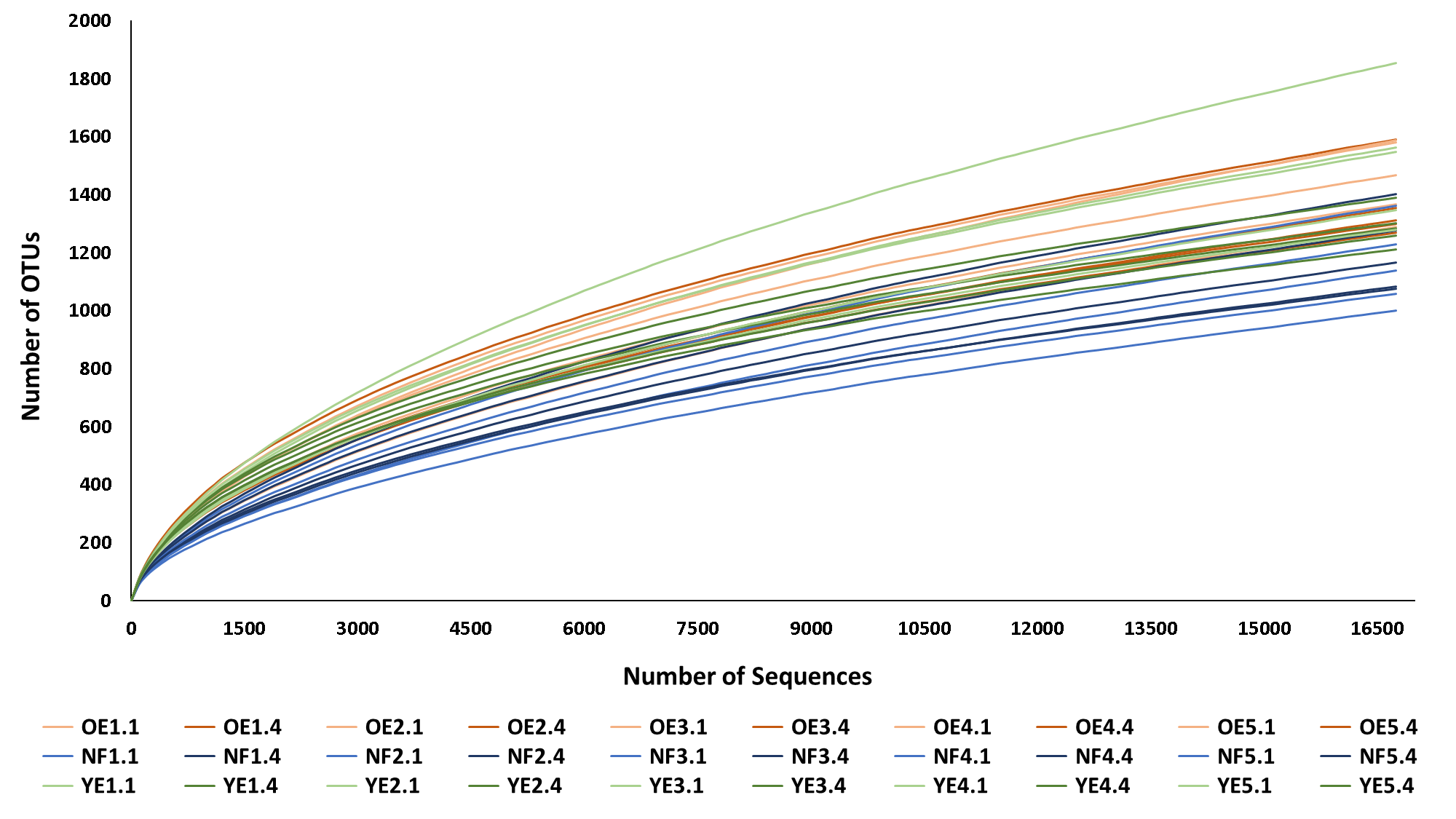
 Supplementary Figure 2 - Rarefaction curves per sample. Old *Eucalyptus* (OE, orange shades), young *Eucalyptus* (YE; green shades), and native forest (NF; blue shades) areas in time 1 (.1; lighter shades) and 4 (.4, darker shades).


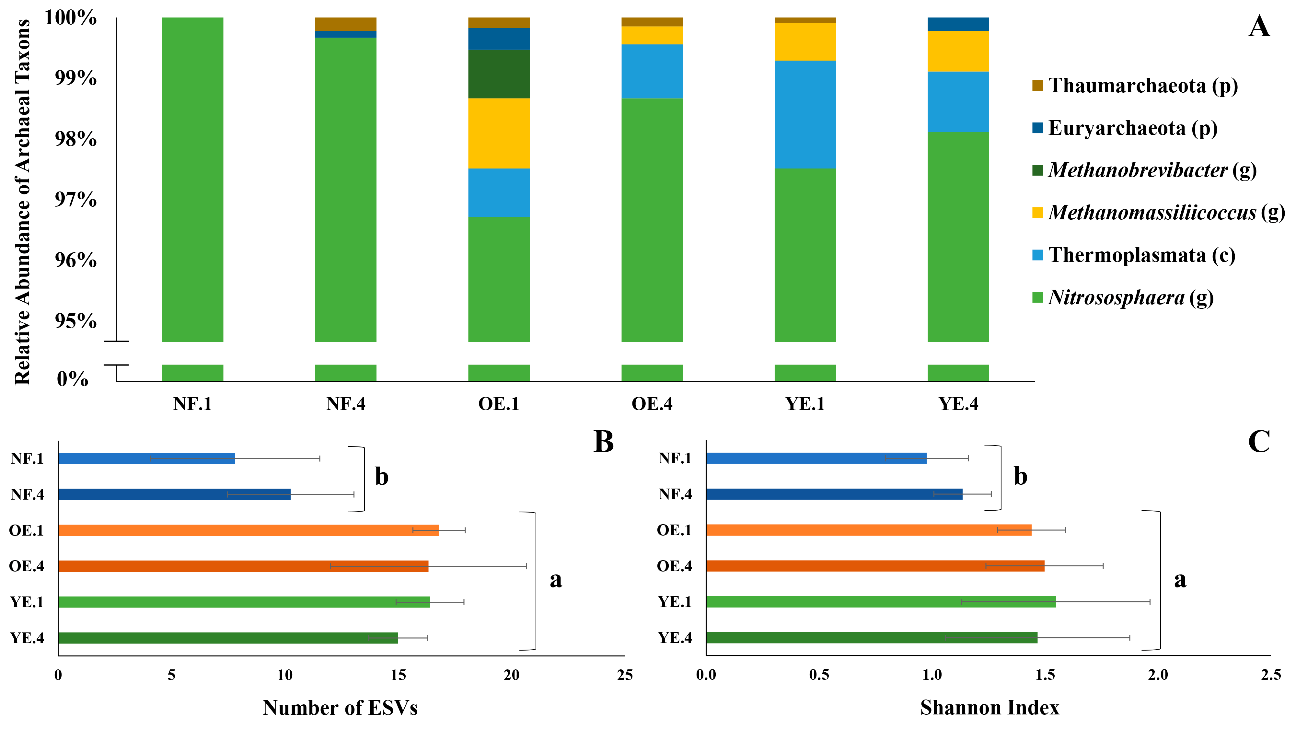


Supplementary Figure 3 – Archaeal composition found in native forest (NF), old *Eucalyptus* (OE), and young *Eucalyptus* (YE) areas at time 1 (.1) and time 4 (.4). Relative abundance of archaeal taxa (A), number of ESVs per treatment (B) and Shannon index (C). Statistical differences (two-way ANOVA followed by Tukey’s test; p < 0.05) among treatments are represented as different letters. Taxonomies were given based on the RDP database with a bootstrap value of 80% (p – phylum, c – class, g – genus).
